# Ground Reaction Forces and Energy Exchange During Underwater Walking

**DOI:** 10.1093/iob/obae013

**Published:** 2024-05-02

**Authors:** K M Gamel, S Pinti, H C Astley

**Affiliations:** Department of Biology, University of Akron, 235 Carroll St., Akron, OH 44325, USA; Naval Undersea Warfare Center, Division Newport, 1176 Howell St., Newport, RI 002841, USA; Department of Biological Sciences, Kent State University, 800 E. Summit St, Kent, OH 44242, USA; Department of Biology, University of Akron, 235 Carroll St., Akron, OH 44325, USA

## Abstract

Underwater walking was a crucial step in the evolutionary transition from water to land. Underwater walkers use fins and/or limbs to interact with the benthic substrate and produce propulsive forces. The dynamics of underwater walking remain poorly understood due to the lack of a sufficiently sensitive and waterproof system to measure substrate reaction forces (SRFs). Using an underwater force plate (described in our companion paper), we quantify SRFs during underwater walking in axolotls (*Ambystoma mexicanum*) and Spot prawn (*Pandalus platyceros*), synchronized with videography. The horizontal propulsive forces were greater than the braking forces in both species to overcome hydrodynamic drag. In axolotls, potential energy (PE) fluctuations were far smaller than kinetic energy (KE) fluctuations due to high buoyant support (97%), whereas the magnitudes were similar in the prawn due to lower buoyant support (93%). However, both species show minimal evidence of exchange between KE and PE, which, along with the effects of hydrodynamic drag, is incompatible with inverted pendulum dynamics. Our results show that, despite their evolutionary links, underwater walking has fundamentally different dynamics compared with terrestrial walking and emphasize the substantial consequences of differences in body plan in underwater walking.

## Introduction

Underwater walking was the behavioral precursor of terrestrial walking and a crucial step in evolutionary transitions from water to land in animals (Edwards 1977; Edwards 1989; Ashley-Ross et al. 2013). Seen as far back as the Cambrian, animals from a wide range of taxa have used substrate interactions to propel themselves within the aquatic environment using modified fins or limbs (Holst and Bone 1993; Kram and Griffin 2000; Lucifora and Vassallo 2002; MacNaughton et al. 2002; Clack 2009; Renous et al. 2011). Underwater walking is known from ancient taxa such as trilobites and stem tetrapods as well as extant salamanders, crocodilians, crustaceans, mammals, and fish (Pridmore 1995; Willey and Blob 2004; Willey et al. 2004; Clack 2009; Coughlin and Fish 2009; Macesic and Kajiura 2010; Jamon et al. 2011; King et al. 2011; Renous et al. 2011; Kawano and Blob 2013; Pierce et al. 2013; Flammang et al. 2016; Dickson and Pierce 2018; Hunyadi et al. 2020). Underwater substrate interactions create opportunities for concealment, feeding, controlled locomotion, and resting in flow (Martinez 1996). Exploring cluttered environments away from the negative impacts of competitors and predators offer new niches (Edwards 1977) and opportunities for species to evolve morphotypes (modified fins and limbs) specific for this ecological niche (Daeschler et al. 2006; Hsieh 2010). The diversity of modified limbs/fins demonstrate how substrate interaction and environmental topography provide opportunity for morphological innovation (Martinez 2001; Ashley-Ross and Bechtel 2004; Ashley-Ross et al. 2009; Ashley-Ross et al. 2013; Pronko et al. 2013; Petti et al. 2014).

Underwater walkers face a unique mix of fluid and substrate reaction forces (SRFs) (Martinez 1996). The fluid forces acting on the body are buoyancy, drag, lift, and the added mass effect (Maude and Williams 1983; Martinez 2001; Lim and DeMont 2009). Buoyancy counteracts gravity, supporting the majority of an animal's body weight (Zug 1971; Peterson and Gomez 2008; Alam et al. 2015). An aquatic animal can modify its buoyancy, often by changing the gas volume in the lungs or the swim bladders (Zug 1971; Peterson and Gomez 2008). Most animals support 4–20% of their gravitational body weight during underwater walking (Pond 1975; Dickson and Pierce 2018). Aquatic animals can also modify their body density with increased compact bone thickness and gastroliths (Pond 1975; Clarac and Cruse 1982; Maude and Williams 1983; Wall 1983; Houlihan and Innes 1984; Schreiner 2004; Adamowicz et al. 2008; Lim and DeMont 2009; Tanacredi etal. 2009; Chabot and Watson 2010). During locomotion, the viscosity and dynamic pressure of the fluid environment impose hydrodynamic drag, which opposes motion. Drag force is affected by speed, surface area, and body form (streamlined vs bluff/blunt bodies). The body form can also change the direction of the passing water, which generates negative or positive lift (Martinez et al. 1998). A boundary layer accumulates around the surface of the moving body, which not only causes the skin friction component of hydrodynamic drag (Lauder 1996; Plotnick and Baumiller 2000; Taft et al. 2008) but also requires that an accelerating animal to impart momentum to both its own mass and the mass of fluid in the boundary layer, known as the added mass effect (Wang et al. 1996). Finally, like terrestrial walkers, underwater walking generates propulsive force by pressing posteriorly against the substrate, resulting in equal and opposite reaction forces on its limbs which push the animal forward (Dickinson et al. 2000).

Prior studies on underwater walking have examined the gait cycle, speed, and body and limb kinematics (Ashley-Ross and Bechtel 2004; Starke and Clayton 2015; Chellapurath et al. 2020). However, data on the kinetics (forces acting on the body) of this locomotor mode have been scarce and limited due to the complications and expense of force- sensing technology in water (Clarac and Cruse 1982; Klarner and Barnes 1986; Jamon et al. 2011). One prior study on flying gurnards used a calibrated photoelastic substrate to gather total force magnitude (Jamon et al. 2011) but the directional components of the force were not quantified. Another study used an octopus-inspired legged robot and calculated force applied to substrate using a mathematical model (Calisti et al. 2015), but only peak forces were reported and the applicability to biological underwater walking remains unclear. Two prior studies report single leg forces in crustaceans (Clarac and Cruse 1982; Klarner and Barnes 1986). One gathered single-leg force data in the horizontal plane only with strain gauges affixed to the limb tip (Clarac and Cruse 1982), while another used a small platform with two load cells (Klarner and Barnes 1986). Single leg forces can allow calculation of joint moments if combined properly with kinematic data (Winter 2009), while whole body kinetics can reveal the effect of the SRFs on the whole body motion, particularly with regard to pendular energy exchange seen in many terrestrial walkers (Dickinson et al. 2000; Biewener and Patek 2018). Other authors note the importance of gathering SRFs during underwater walking, especially for comparing patterns on land vs water (Martinez et al. 1998; Dickinson et al. 2000). These different environments are expected to impose different loads on the limb and body, and thus impose different selective pressures on morphology. Thus, gathering more complete and detailed SRFs of underwater walking will fill critical gaps in our understanding of the evolutionary transition from water to land (Koehl 1996; Dickinson et al. 2000). Our recently developed underwater force plate is large enough to gather whole body forces in three axes independently while retaining sufficient sensitivity to detect the small forces seen in underwater walking (Gamel et al. 2024). When combined with synchronous video, this allows quantification of both SRFs and kinetic and potential energy (PE) fluctuations of the whole body during continuous locomotion (Gamel et al. 2024).

The objective of this investigation was to quantify the dynamics of underwater walking by collecting whole body SRFs of axolotls (*Ambystoma mexicanum* [Shaw and Nodder 1798]) and Spot prawn (*Pandalus platyceros* [Brandt 1851]) using a custom-built underwater force plate with synchronous video. Digitizing videos allowed us to quantify the center of mass (CoM) velocity and position, as well as footfalls, to compare force production with kinematics (Starke and Clayton 2015) and calculate the kinetic energy (KE) and PE changes during locomotion. Salamanders are a model system for underwater walking and axolotls are readily available, large, and regularly perform underwater walking (Pierce et al. 2012; Pierce et al. 2013; Dickson and Pierce 2018). Many crustaceans spend much of their adult lives moving on benthic substrates via their modified limbs and their different body plan and density provides a mechanical contrast to axolotls.

We hypothesize that, in contrast to terrestrial walking, the average propulsive force of underwater walking would be greater than the average braking force due to the drag from the viscous environment. We also hypothesize that the peak fore-aft forces will be similar in magnitude to the peak vertical force, due to the combination of drag and buoyant support, in contrast to terrestrial walking, in which vertical forces are much higher to support body weight. We predict minimal energy exchange between KE and PE due to buoyancy reducing the PE but leaving the KE unaffected, though the higher density of prawns will increase their PE. Finally, axolotls may show more variability in forces and speed than prawns due to phase offsets in limb use in prawns, as seen with gurnard fin rays (Wings 2007; Jamon et al. 2011). We wish to emphasize that we do not intend to draw adaptive conclusions from differences between these two species (Garland and Adolph 1994) but instead to test for similarities predicted by universal physical laws which should be present regardless of phylogeny or anatomy.

## Methods

### Animals

Five axolotls were purchased from the Ambystoma Genetic Stock Center and housed in 55-gallon tanks at a water temperature of 16°C at the University of Akron (UA). They were fed 2 times a week and were on a 12:12 light schedule. All axolotls were adults with a total (body and tail) length of 25.7 ± 1.7 cm (mean ± std. dev) and a mass of 112.7 ± 10.8 g (terrestrial weight = 1.1 ± 0.1 N). The underwater weight was 26.9 ± 11.0 mN, and was 0.024 denser than freshwater, therefore the body is approximately 97.6% supported by buoyancy. Underwater weight was calculated as the average vertical weight over an entire locomotor trial. Axolotl procedures were approved under UA IACUC protocol 22–05-04-AAC.

Six spot prawns (*P. platyceros*) were trolled in the Salish sea and stored in 55-gallon tank at 16°C in salt water at Friday Harbor Labs (FHL). The prawn had an average mass of 20.3 ± 1.58 g, corresponding to a terrestrial weight of 199.07 ± 15.49 mN, and a total body length of 13.3 ± 0.3 cm. Underwater weight was an average of 13.9 ± 2.1 mN and was 0.068 denser than saltwater, therefore the body is approximately 93.2% supported by buoyancy. Underwater weight was calculated as the average vertical weight over an entire locomotor trial. Prawn procedures were approved under University of Washington IACUC- 4308–4.

#### Locomotion tests

This study utilizes a custom designed force plate, described in the accompanying methods paper (Gamel et al. 2024) to gather SRFs. Locomotion tests were performed at two locations (UA and FHL) with similar force plates constructed at each location. The force plate sensing area was 30.5 × 15.3 cm (UA) or 22.0 × 14.0 cm (FHL) and consisted of custom 3D printed load cells with custom circuitry. We used a plastic egg crate platform consisting of a grid of 1 cm square holes open on the top and bottom during trials. Preliminary experiments with a solid platform detected the forces from water movements (Gamel et al. 2024), obscuring the signal from SRFs. Using the plastic grid alleviated the conflicting signal received from the water motion produced by the animal, while also providing grip points on the substrate and reducing foot slip. The resolution of the raw signal for the force plate is 5 mN, but once low-pass filtered, the resolution is 1 mN. GoPro Hero 6 Black cameras recorded lateral (UA & FHL) and dorsal (UA only) views at 60 frames per second (Supplementary Videos).

**Fig. 1:**
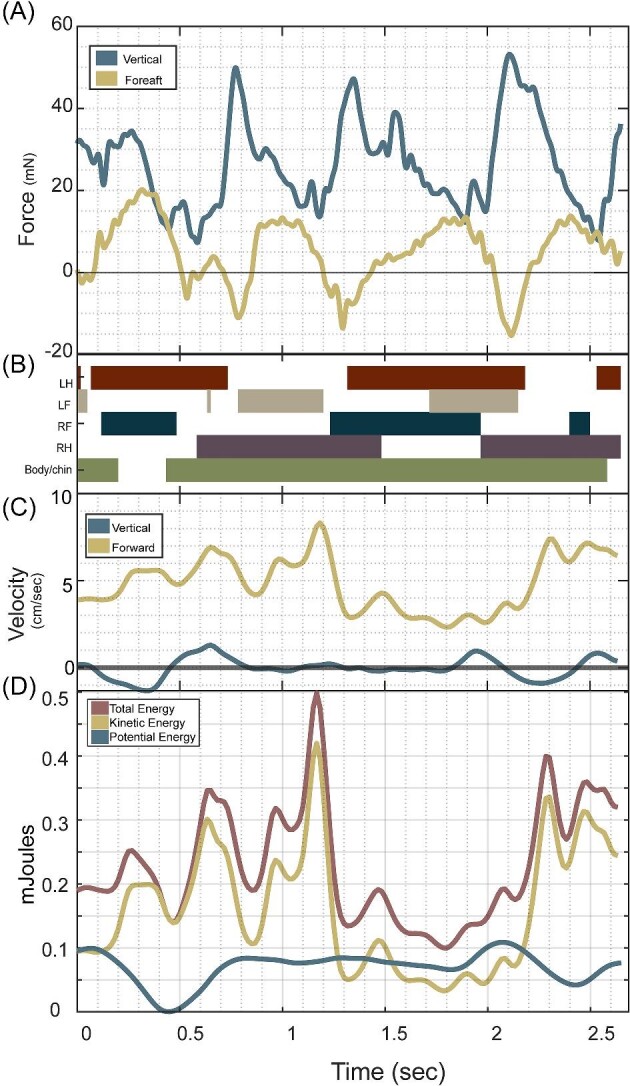
An example trial of axolotl walking underwater, displaying a more regular movement pattern (Supplements; movie 1 and 2). (A) Substrate reaction forces (SRFs) over time (expressed in milliNewtowns (mN) on the left axis and percent of terrestrial body weight on the right axis) in the vertical (blue) and fore-aft (yellow) directions, with positive values of each designating body support and propulsive force, respectively. (B) Gait diagram displaying contacts of all four limbs, as well as body and chin contact with the substrate. (C) Velocity (cm/s) of the proxy center of mass (CoM) point in the vertical (blue) and horizontal (yellow) directions. (D) Energy (µJ) over time, including kinetic (yellow), potential (blue), and total (red). In (C) and (D), a decrease in forward velocity and kinetic energy (KE) occurs at 1.25–2.25 second due to a stumble, which is then followed with an “early” footfall timing of the left front to catch itself and attempt to continue forward.

**Fig. 2:**
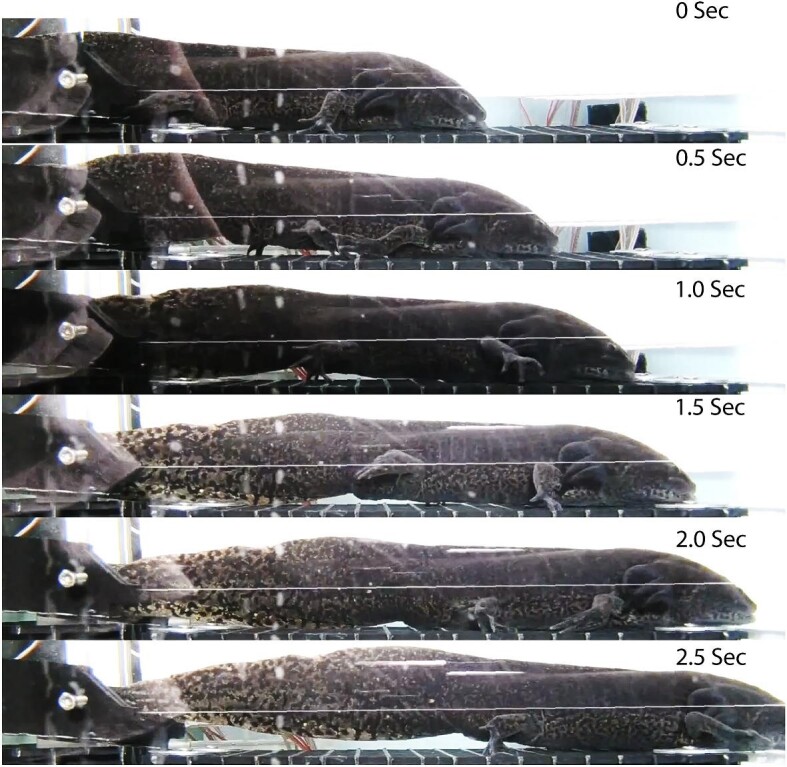
Still video photos of figure 1 (Supplements; movie 1 and 2). *Note: At 1 s, the experimenter moved in front of the experimental light.

We gathered reaction forces from 20 axolotl trials and 28 prawn trials performing underwater walking. Axolotls and prawns moved across the force platform in a 40-gal (UA) or 25-gal (FHL) aquarium filled with 40% Holtfreter's solution (UA) or saltwater (FHL) at 16°C. We gathered vertical, fore-aft, and medio-lateral forces synchronized with video; medio-lateral forces were excluded from axolotl data due to sensor issues arising from corrosion around an inadequate silicone seal. The trackway included the force plate, sidewalls, a starting platform, and an ending platform. Because the force plate was sufficiently sensitive to detect small water currents (Gamel et al. 2024), motion of the experimenter's hands had to be minimized. Before a series of trials began, we zeroed and checked the calibration of the force plate before recording data. During each trial at both locations, a small weight was placed onto the plate to synchronize the force recordings and video, along with checking the calibration of the force plate. Trials that drifted more than 0.4 V in either setup were discarded, as were trials where signals from water disturbances or swimming motions from the tail or pleopods were apparent (Figs. 1 and 2).

We used a NIDAQ USB-6002 (National Instruments Corporation, Austin, TX, USA) and IGOR Wavemetrics (WaveMetrics, Portland, OR, USA, Version7) (UA) or MATLAB (MathWorks, Natick, MA, USA) (FHL), to gather directional force data at 1000 Hz, with positive values of each representing SRFs supporting the body and imparting propulsion, in the vertical and fore-aft directions, respectively. Forces were filtered using a low pass filter with 101 coefficients in IGOR or 60 coefficients in MATLAB, with cutoff frequencies starting at 10 Hz and complete rejection at 20 Hz. Videos were synchronized with each other (UA) and the force plate (UA & FHL) in customized code in MATLAB via the timing of a weight which was dropped and removed, after which the data was cropped to the times the animal was completely on the force plate (i.e., limbs were no longer in contact with the starting and ending platforms). To determine our calibration matrix for force data, we used least squares solution (scipy.linalg.lstsq) in Python (Python, Lacombe, LA, USA) using the change of voltage and the known mass (10, 5, and 2 g) in all 6 directions. The mean residual was ±2.3 mN and an *r*^2^ = 0.993 for the UA system, and ±2.1 mN with an *r*^2^ = 0.985 for the FHL system. Our calibration matrix in grams was multiplied by the voltage output over time and multiplied by 9.8 to attain weight or force in milliNewtons (also seen in Biewener and Full 1992; Gamel et al. 2024).

Axolotls were placed on the starting platform and encouraged to move across the force plate and onto the ending platform by slightly pinching the tail (Supplementary Videos). A maximum of 30 trials (including failures) were recorded per axolotl per day to minimize stress on the axolotls and prevent fatigue. The axolotls were never away from the vivarium for longer than 2 h and rested for at least a minute between trials. Prawn continuously walked back and forth for 60–90 min with minimal experimenter intervention and no signs of fatigue, and data from all prawn was gathered over the course of two days.

For axolotls, we digitized the tip of the snout and a proxy CoM point for all frames using DLTdv8 (Hedrick 2008). We also used intermittently digitized points to record timing and duration of foot contacts, chin/body contacts, calibration weight contact and removal, as well as two points in the environment separated by a known distance to convert pixels to cm. The proxy CoM point was located approximately midway between the pectoral and pelvic girdles; as the axolotls showed minimal lateral flexion of the body or rotation in any axis, this serves as a reliable CoM proxy for calculating velocity and KE and PE fluctuations, as these are only dependent upon the changes in CoM position over time. To quantify motion of the prawn, we digitized three points for every frame within each trial, aided by natural markings of the animal: below the eye at the start of the white line, the white middle dot on the carapace, the white dot on the tail. We also placed a digitized point in the frame when the weight came off the force plate, and at two reference points to convert pixels to centimeters. The middle white dot on the carapace was a proxy for CoM and was used to calculate velocity and KE and PE fluctuations. Manual placement of the camera occasionally produced a tilted image, which was corrected using a coordinate transformation. We used the coordinates of two reference points on the force plate to determine the angle of camera tilt (*q* = tan^−1^ [*x*_2_ − *x*_1_]/[*y*_2_ − *y*_1_]), and multiplied the untransformed coordinate system by the rotation matrix [cos (q), sin (q); −sin (q), cos (q)] to determine the *X* (horizontal) and *Y* (vertical) position values of the proxy CoM point. For both axolotls and prawns, we smoothed position values of the CoM digitized point using cubic smoothing spline method for *x, y*, and *z* coordinates independently and then calculated variables in MATLAB.

#### Variables

Due to high variability of footfall patterns in axolotls, we could not delineate clear cycles (see Results), precluding traditional gait analysis or partitioning the data beyond the level of the whole trial. Although the prawns showed more regular limb patterns, we used a similar whole-trial analysis to facilitate comparisons between the taxa. From the force data of both the prawn and the axolotl, we calculated the mean, maximum, and minimum for both the vertical and fore-aft forces, and for the medio-lateral forces for spot prawn. For fore-aft forces, we also computed the mean purely propulsive (fore) and purely braking (aft) forces for both animals. These were also expressed as the magnitude and angle of the reaction force vector over time, with the former computed as based only on fore-aft and vertical forces (to reflect the lack of medio-lateral forces for the axolotl and facilitate comparisons) and the latter computed as the arccosine of the fore-aft force divided by total force; we report both the mean for each trial (to assess propulsive vs braking) and the fluctuation (see below). Due to the small magnitudes of the lateral forces in prawns, results for total forces including and without lateral forces were minimally different.

From kinematic data, we quantified the mean, maximum, and minimum vertical, horizontal, and total velocity of the CoM point. Kinetic energy was calculated as typical (0.5 * mass * velocity^2^). However, PE represents the work done to move an object against a conservative force, and thus must account for buoyant support of the media or it will over-predict the energy transformed into KE when an object in a fluid is falling (even in the absence of drag). Thus, to calculate PE, we used underwater weight * height, with underwater weight defined as the mean average vertical force of each trial to account for buoyancy. We calculated the mean, maximum, minimum, and range of values of KE and PE. For the axolotls, we also computed the number of footfalls per limb in the trial and a non-cyclic approximation of duty factor, which was calculated by dividing total duration of foot contact throughout the trial by total trial duration, averaged across both forelimbs and both hindlimbs.

To remove the influence of trips and falls, we also calculated the standard deviation of the total velocity, the force magnitude and angle, and the KE and PE within each trial, such that 95% of values for a given trial fall within plus or minus two standard deviations. Thus, we report both the maximum minus minimum value (“range”) and four times the standard deviation (“fluctuation”) of each variable. To facilitate comparisons of variability between the species, we also computed the coefficient of variation (=standard deviation/mean) for each trial for the above variables.

The existence of oscillations of kinetic and PE, even if similar in magnitude, does not necessarily indicate pendular energy exchange as seen in terrestrial walking; such changes must be out of phase or otherwise complementary, with increases in one corresponding to decreases in the other. To assess the potential for pendular energy exchange, we calculated the rate of change of kinetic and PE within each trial. In an ideal pendulum, the rate of increase of KE would equal the rate of loss of PE, and vice versa; when graphing the rates of change of each relative to the other, this would be a diagonal line with a slope of –1 passing through the origin. We categorize every instantaneous pair of powers as Purely Added Work (positive power for both kinetic and PE), Pure Losses (negative power for both), Potential Exchange With Added Work (one power is positive and another negative, but the sum is greater than 0 (above the ideal pendulum line)), and Potential Exchange With Loss (one power is positive and another negative, but the sum is less than 0 (below the ideal pendulum line). Unfortunately, partitioning out how much energy is exchanged between kinetic and PE as opposed to generated by muscular work or lost to drag is beyond the scope of this paper.

## Results

**Fig. 3:**
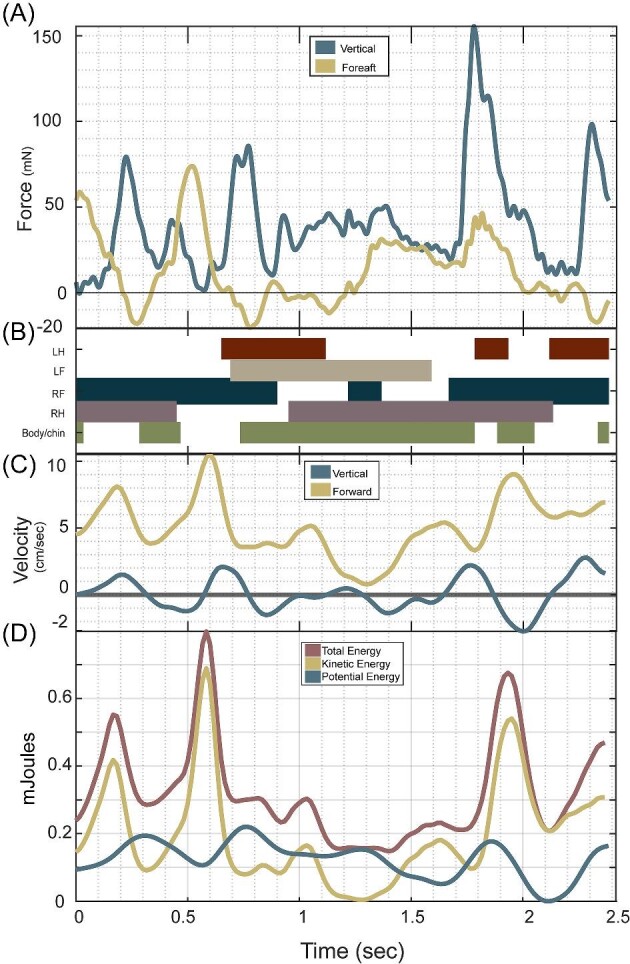
An example trial of axolotl walking underwater, displaying an irregular movement pattern (Supplements; movie 3 and 4). A “stumble” occurs at 1.7 s, consisting of a left forelimb impact followed quickly by a body impact. (A) Substrate reaction forces over time (expressed in milliNewtowns [mN] on the left axis and percent of terrestrial body weight on the right axis) in the vertical (blue) and fore-aft (yellow) directions, with positive values of each designating body support and propulsive force, respectively. (B) Gait diagram displaying contacts of all four limbs, as well as body and chin contact with the substrate. (C) Velocity (cm/s) of the proxy CoM point in the vertical (blue) and horizontal (yellow) directions. (D) Energy (µJ) over time, including kinetic (yellow), potential (blue), and total (red). In (C) and (D), a decrease in forward velocity and KE occurs at to 1.75 s due to a stumble, which is then followed with both left limbs down at the same time and a misstep in the swing forward phase of the right front limb.

**Fig. 4:**
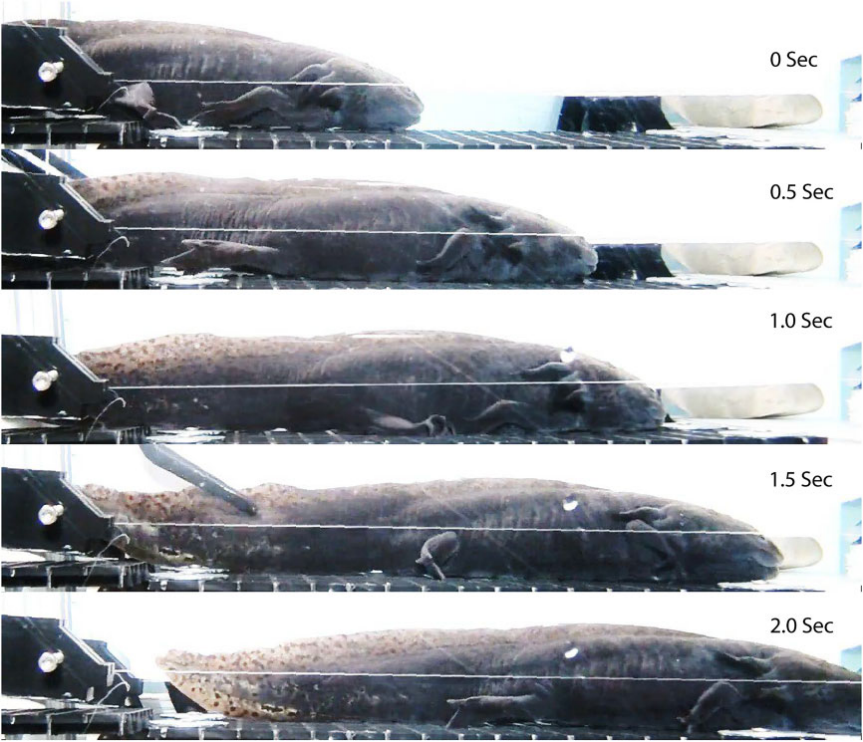
Still video photos of figure 3 (Supplements; movie 3 and 4).

**Fig. 5:**
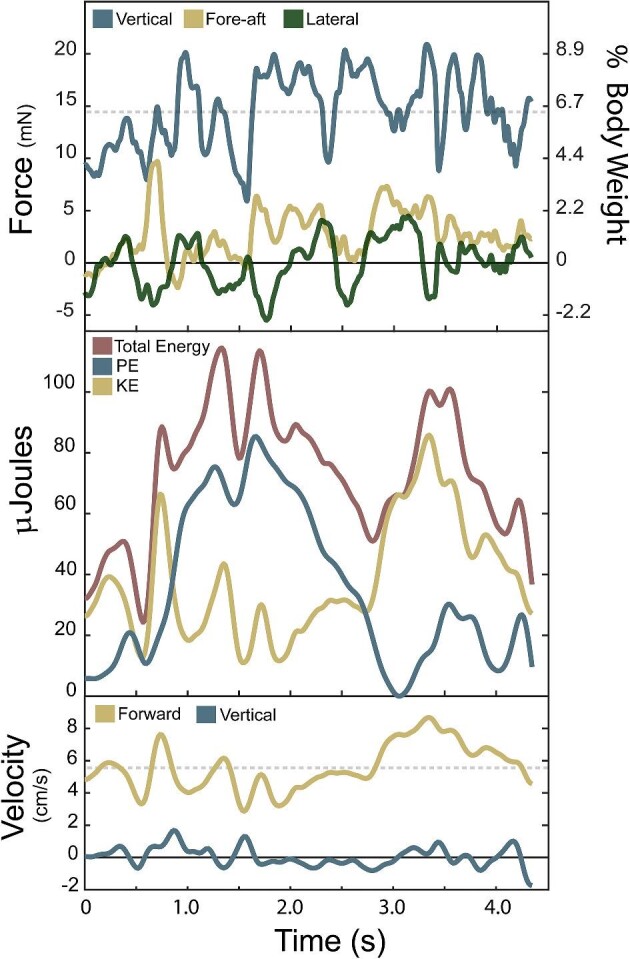
Walking force, energetic fluctuation, and directional velocity of a single prawn trial. Dotted lines represent the mean value of vertical force and forward velocity (Supplements; movie 5)). (A) The SRFs and land body weight percentage of vertical, fore-aft, and lateral forces of prawn walking across a force plate. Negative fore-aft is the braking force and positive is propulsive. (B) Total, potential, and KEfluctuations over the trial. (C) The forward and vertical velocity of the CoM over the trial of the same trial seen in Fig. 4A. The total energy is the sum of kinetic and potential energy (PE).

**Fig. 6:**
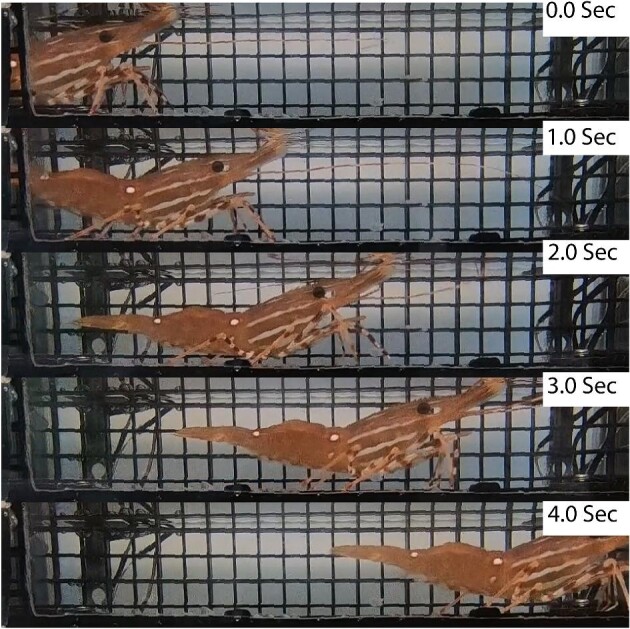
Still video photos of figure 5 (Supplements; movie 5).

Axolotls had an average total velocity of 7.7 ± 3.0 cm/s (mean ± std. dev.) (Figs. 3 and 4, Table 1), giving a Reynolds number of approximately 1.8 × 10^4^. Axolotl forelimbs were in contact with the substrate for 36.3 ± 0.1%, while hindlimbs were in contact for 51.2 ± 0.1% (Figs. 3–6, Table 1). Axolotl footfall patterns and forces were highly variable and did not show consistent gait patterns during a trial, and this variability appeared to increase with speed (Figs. 3–6). Velocity varied within a trial as well as between trials, with a mean peak total velocity of 15.0 ± 6.1 cm/s and minimum of 2.6 ± 2.2 cm/s (Table 1, Figs. 3 and 4). We further quantified this variability of the total velocity within a trial as the fluctuation (11.0 ± 3.9 cm/s) and coefficient of variation (0.38 ± 0.13).

**Table 1:** Kinematic, kinetic, and energetic variables calculated.

Variable	Axolotl	SD	Prawn	SD
Average velocity (cm/s)	7.7	3.0	4.4	1.3
Vertical velocity (cm/s)	0.19	0.39	0.02	0.15
Forward velocity (cm/s)	7.6	2.7	4.4	1.3
Peak total velocity(cm/s)	15.0	6.1	7.4	2.3
Min total velocity (cm/s)	2.6	2.2	1.6	1.3
Velocity fluctuations (SD*4)	11.0	3.9	5.4	2.3
Coefficent variation velocity (cm/s)	0.38	0.13	0.32	0.14
Average propulsive force (fore) (mN)	11.84	6.57	2.39	1.13
Average braking force (aft) (mN)	−7.30	5.47	−1.11	0.83
Average fore-aft force (mN)	5.30	3.96	1.75	1.45
Average fore peak force (mN)	30.80	17.22	7.03	2.51
Average aft peak force (mN)	-24.95	17.83	1.49	1.51
Average vertical force (mN)	26.93	11.01	13.88	2.07
Average vertical peak force (mN)	70.25	34.38	22.05	4.17
Average vertical min force (mN)	1.47	7.93	5.21	3.27
Vertical force magnitude (max–min) (mN)	68.78	35.27	15.75	6.50
Fore-aft magnitude (max–min) (mN)	55.75	27.40	8.82	4.53
Mean total force (mN)	30.66	11.92	14.14	2.10
Mean vector force angle (degrees)	76.68	9.51	82.00	4.70
Average lateral force (mN)	–	–	0.24	1.91
Lateral force range (max–min)	–	–	8.75	4.60
Total force magnitude fluctuations	57.47	27.57	13.15	4.86
Coefficent variation total force magnitude	0.48	0.15	0.23	0.08
Vector force fluctuations	100.9	37.7	31.1	10.8
Coefficient of variation vector force	0.340	0.160	0.096	0.034
Kinetic energy range (max–min) (µJ)	1400.02	1218.54	56.60	48.20
Potential energy range (max–min) (µJ)	197.01	144.87	139.30	58.00
KE fluctuations (SD*4) (µJ)	1191.44	944.48	56.95	41.72
PE fluctuations (SD*4) (µJ)	217.76	176.32	139.80	68.36
Average KE (µJ)	424.47	311.57	24.70	13.40
Average PE (µJ)	110.81	92.55	74.60	37.90
Peak KE (µJ)	1463.27	1275.52	65.55	50.44
Peak PE(µJ)	197.01	144.87	139.33	57.97
Average total energy (µJ)	532.2	359.5	99.3	42.2
Total range energy (max–min) (µJ)	1379.7	1136.4	127.6	64.3
Total energy fluctuations (SD*4) (µJ)	1149.8	859.3	137.1	65.8
Pure positive power %	24.7	–	23.6	–
Pure negative power %	22.1	–	22.0	–
Potential pendular exchange plus work %	26.0	–	27.7	–
Potential pendular exchange with loss %	27.2	–	26.7	–

**Fig. 7:**
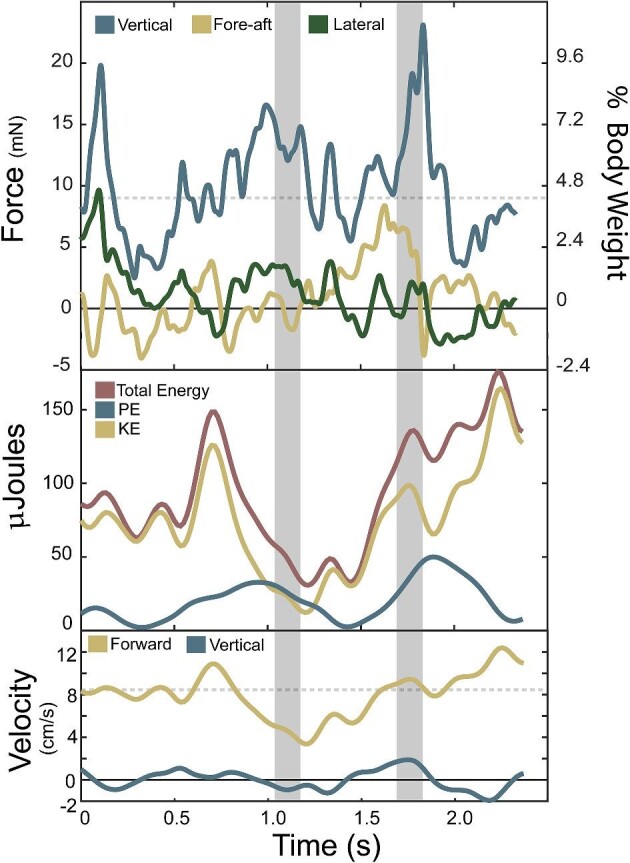
Prawn walking (gray shaded region) and using swimmerets (white region) presenting force, energetic fluctuation, and directional velocity of a single prawn trial (Supplements; movie 6). (A) The SRFs and land body weight percentage of vertical, fore-aft, and lateral forces of prawn walking across a force plate. Negative fore-aft is the braking force and positive is propulsive. (B) Total, potential, and KE fluctuations over the trial. (C) The forward and vertical velocity of the CoM over the trial of the same trial seen in Fig. 7A. The total energy is the sum of kinetic and PE.

Prawn moved rapidly across the force plate, with an average total velocity of 4.4 ± 1.3 cm/s (mean ± std. dev.) (Fig. 7), giving a Reynolds number of approximately 5.2 × 10^3^. While animals moved horizontally, they would occasionally raise or lower their body during the trial, then continue to move forward with their new posture. If the animal did not revert to its original posture by the end of the trial, it would result in a net positive or negative vertical velocity for that trial. Velocity varied within a trial as well as between trials, with a mean peak total velocity of 7.4 ± 2.3 cm/s and minimum of 1.6 ± 1.3 cm/s (Table 1, Fig. 7). We further quantified this variability of the total velocity within a trial as the fluctuation (5.4 ± 2.3 cm/s) and coefficient of variation (0.32 ± 0.14).

For axolotls, the average propulsive (fore) force of 11.84 ± 6.57 mN was greater than the average braking (aft) force of −7.33 ± 5.47 mN (Figs. 3 and 4, Table 1). The axolotl's average fore-aft force was 5.30 ± 3.96 mN and has an average fore peak is 30.80 ± 17.22 mN and aft minimum is −24.95 ± 17.83 mN (Figs. 3 and 4, Table 1). Vertical and fore-aft forces had a similar range (maximum minus minimum) in axolotls with 68.78 ± 35.27 mN for vertical and 55.75 ± 27.40 mN for fore-aft (Figs. 3 and 4, Table 1). The axolotls had a mean SRF vector angle of 76.68° ± 9.51° (where purely propulsive is 0° and vertical is 90°) (Table 1). Total force magnitude showed fluctuations of 57.47 ± 27.57 mN and a coefficient of variation of 0.48 ± 0.15, while force angle showed fluctuations of 100.9° ± 37.4° and a coefficient of variation of 0.34 ± 0.16.

For prawns, the average propulsive (fore) force of 2.39 ± 1.13 mN is greater than the average braking (aft) force of −1.11 ± 0.83 mN (Fig. 7). The prawn's average fore-aft force was 1.75 ± 1.45 mN and has an average fore peak of 7.03 ± 2.51 and aft minimum of 1.49 ± 1.51 (Fig. 7). Vertical and fore-aft forces had a similar range (maximum minus minimum) in prawns with 15.75 ± 6.50 mN for vertical and 8.82 ± 4.53 mN for fore-aft (Fig. 3, Table 1). The prawns had a mean SRF vector angle of 82.0° ± 4.7° (Table 1). Total force magnitude showed fluctuations of 13.15 ± 4.86 mN and a coefficient of variation of 0.23 ± 0.08, while force angle showed fluctuations of 31.1 ± 10.8 and a coefficient of variation of 0.096 ± 0.034.

During underwater walking in axolotls, the KE range is much higher than PE, 1400.02 ± 1218.54 µJ vs 197.01 ± 144.87 µJ, respectively (Figs. 3 and 4, Table 1). Similarly, the fluctuation of axolotl KE is much greater than axolotl PE, (1191.44 ± 944.48,217.76 ± 176.32 µJ,) (Figs. 3 and 4, Table 1). The axolotls peak KE and PE are 1463.27 ± 1275.52 µJ vs 197.01 ± 144.87 µJ, respectively (Figs. 2–4, Table 1). The axolotls total energy fluctuation is 1149.8 ± 859.3 µJ.

In prawn, the KE and PE ranges are very similar, 56.95 ± 48.20 µJ vs 139.30 ± 58.00 µJ, respectively, as were fluctuations in KE and PE (56.95 ± 41.72 µJ vs 139.80 ± 68.36 µJ, respectively). The prawn's peak KE and PE are 65.55 ± 50.44 µJ and 139.33 ± 57.97 µJ, respectively. The prawn's total energy fluctuation is 137.1 ± 65.8 µJ.

**Fig. 8:**
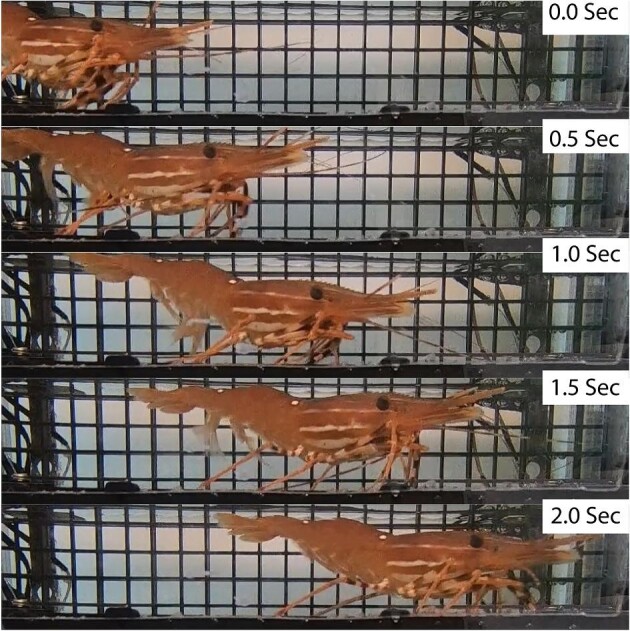
Still video photos of figure 7 (Supplements; movie 6).

The axolotls generated purely positive power for 24.7% of observations (quadrant 1, Fig. 2) and purely negative power for 22.1% of observations (quadrant 4, Fig. 2). Potential pendular exchange with added work occurred in 26.0% (above gold line in quadrant 2 and 4, Fig. 2) of observations, while potential pendular exchange with losses occurred in the remaining 27.2% (below gold line in quadrant 2 and 4, Fig. 2). The prawn generated purely positive power for 23.6% (quadrant 1, Fig. 8) of observations and purely negative powers for 22.0% of observations (quadrant 3, Fig. 8). Potential pendular exchange with added work occurred in 27.7% of observations (above gold line in quadrant 2 and 4, Fig. 8), while potential pendular exchange with losses occurred in the remaining 26.7% (below gold line in quadrant 2 and 4, Fig. 8).

## Discussion

Our study shows minimal use of inverted pendulum mechanics to store energy, a substantial difference from terrestrial walking. Walking terrestrial animals often show successful energy exchange between KE and PE, with similar magnitudes of energy fluctuations occurring out of phase (Cavagna et al. 1976; Cavagna and Kaneko 1977; Heglund et al. 1982; Blickhan and Full 1993; Minetti et al.1994; Muir et al 1996; Cavagna et al. 1997; Farley and Ko 1997; Dickinson et al. 2000; Kram and Griffin 2000; Vogel 2003; Griffin et al. 2004; Sawicki et al. 2009; Winter 2009). Buoyant support poses a particular challenge due to the reduction in PE. This is particularly acute in axolotls, in which high buoyant support reduces PE changes to a small fraction of KE changes, effectively precluding effective pendular energy exchange. However, the increased density of the prawn relative to the surrounding water increases their PE change for the same vertical displacement, resulting in PE fluctuations of similar magnitude to those of KE. While this is a precondition of effective pendular energy storage, this does not necessarily mean that energy from one source is being transferred to another—energy exchange can only occur when the losses in KE are mirrored by gains in PE, or vice versa. We rarely see these symmetrical exchanges (diagonal line on Figs. 2 and 8), with many cases of simultaneous energy increases (i.e., work done on both to accelerate and raise the body, [top right quadrant of Figs. 2 and 8]) and energy decreases (i.e., dissipation of both by drag and falling, [bottom left quadrant of Figs. 2 and 8]). Even when complementary energy increases and decreases occur, most points show additional losses (points below yellow line, Figs. 2 and 8) or additional gains (points above yellow line, Figs. 2 and 8), likely due to drag and muscular work, respectively. Our analysis of energy change over time shows that for both species, approximately equal portions of the data fall into pure added work, pure dissipation, pendular exchange with dissipation, and pendular exchange with added work. Furthermore, very few of the observations fall along the line predicted for an ideal pendulum (Figs. 2 and 8 dark gold line), suggesting that even when pendular exchange could occur, the contribution is likely minimal; the substantial drag incurred, while moving underwater would further reduce the effectiveness of inverted pendulum dynamics. A clear understanding of how energy flows within these systems is beyond the capacity of this study, but the data at hand suggests that pendular energy exchange likely plays a minor role in underwater walking of both species, with active muscular work and hydrodynamic drag dominating. Examination of energy flow in robotic underwater walkers with bio-inspired gaits, as in (Calisti et al. 2015; Picardi et al. 2018), may provide a vital tool by allowing quantification of motions, drag, and actuator work and their relative changes.

How animals made the transition from underwater walking to pendular terrestrial walking remains unknown, and may depend upon body size, density, or the consequences of partial submersion. Since buoyancy affects the vertical load, animals that have higher density may encounter larger PE fluctuations and may be more in range with the KE fluctuations, as seen in prawns. Increased body size will increase mass, and thus the relative role of inertia, more rapidly than surface area, which determines drag and associated hydrodynamic forces, suggesting that much larger animals (e.g., early tetrapods) may have been able to use pendular energy exchange more effectively. However, this would be contingent upon having joints and muscles capable of generating sufficient torques. Further comparative studies will establish a better understanding of the role of PE in underwater walking across different taxa and kinematic strategies.

The forces we measured during underwater walking differed substantially from patterns typically seen in terrestrial walking, with greater propulsive force than braking force and vertical forces similar in magnitude to fore-aft forces confirming our hypotheses (Figs. 3, 4, and 7). Terrestrial walking at a steady overall speed has a net zero fore-aft force over a stance phase (Wang et al. 2003; Rubenson et al. 2004; Kuo 2007; Wannop et al. 2012). However, hydrodynamic drag exerts a braking force on any object moving within a fluid, which requires net propulsive work to maintain speed and correspondingly can reduce or eliminate the need for an animal to perform braking work. Adult human walkers partially submerged in water (above waist) completely lack braking forces and show net propulsive forces essential to maintain forward velocity to offset drag (Barela et al. 2006). Our results are less dramatic, with braking forces being still present but lower than propulsive forces (Figs. 3 and 4), which is consistent with the more streamlined body form of the axolotl and prawn compared to an upright, wading human. The observed prawn forces are also consistent with single foot reaction forces from (Klarner and Barnes 1986), despite the larger mass of their crayfish. Another major difference is the lower vertical forces during underwater walking, with magnitudes broadly similar to fore-aft forces (Figs. 3 and 4), compared to the much higher vertical ground reaction forces needed to counteract gravity in salamanders and other terrestrial walkers (Cavagna et al. 1997; Sheffield and Blob 2011; Kawano and Blob 2013; Kawano et al. 2016). In axolotls, the vertical and force-aft forces are nearly equal, whereas in the prawn, the vertical forces were substantially greater, though not quite to the degree seen in terrestrial walkers. This is consistent with the differences in buoyant support between the species, with the axolotl being almost entirely buoyantly supported (97%) vs the denser prawn (93%). Thus, the denser crustaceans might experience substantial differences in not only magnitude, but patterns of limb joint loading compared to the axolotls, though testing this would require single-foot forces and limb kinematics from both. These results show that underwater walking is subject to substantially different force magnitudes and orientations compared with terrestrial walking, which in turn imposes different mechanical demands upon the musculoskeletal system of the appendage. These different demands can lead to differences in the selective pressures on the limbs of aquatic organisms, and well as relaxation of these pressures due to the lower overall forces, which may in turn contribute to a greater diversity of functionally useful limb shapes (Young and Blob 2015; Young et al. 2017).

The limited force data available from prior studies gives us some insights into our results, via comparisons with species of highly different morphologies (Clarac and Cruse 1982; Klarner and Barnes 1986; Jamon et al. 2011). Two papers studied crayfish and lobsters, which use eight walking legs (Clarac and Cruse 1982; Klarner and Barnes 1986), while (Jamon et al. 2011) studied gurnards, a bony fish which uses three pairs of modified fin rays to walk, in contrast to our taxa, which use four and six walking limbs. Although precise comparisons are difficult due to methodological and taxonomic differences, these prior studies (Klarner and Barnes 1986; Jamon et al. 2011) show similar force magnitudes for underwater walkers of similar masses. All three showed simple patterns of force change over time for individual fin rays or legs (Clarac and Cruse 1982; Klarner and Barnes 1986; Jamon et al. 2011), but the varying force magnitude combined with offset appendage timing was shown to result in a less variable net force in (Jamon et al. 2011). This variability is similar to our recorded whole-body forces, suggesting that single-leg force recordings from axolotls may yield clearer patterns of force change over time.

**Fig. 9:**
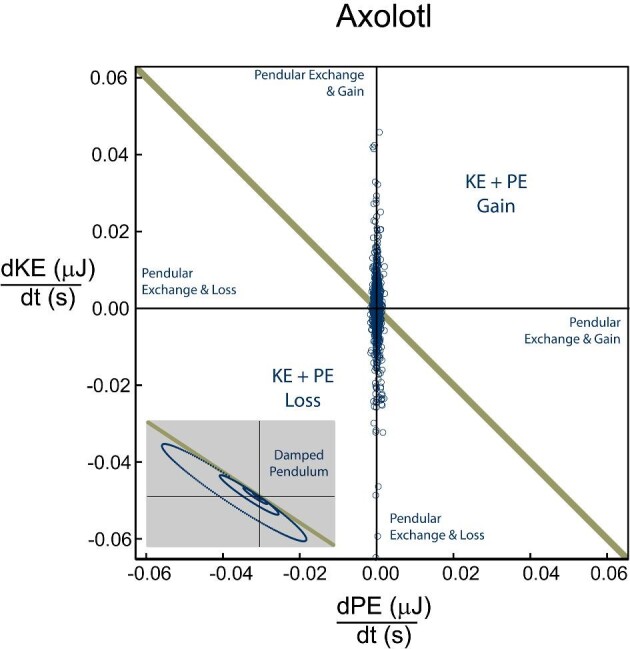
Rates of energy change in Axolotl, showing the relationship between the rate of change (power) for potential and KE in all trials of underwater walking in axolotls. For a perfect pendular system, the results fall on the diagonal line (left top corner to bottom left corner of figure) while our data sits mostly along the y-axis. Due to the minimal PE fluctuations, pendular exchange is minimal. Data points are roughly evenly distributed between pure loss, pure work, and low power pendular exchanges with losses or gains. The bottom left is an example of a dampened pendulum with no energy added to the system.

During our trials, axolotls showed very high variability in footfall patterns and other kinematics, to the point of obscuring any cyclicality in movement patterns, particularly at higher speeds (Figs. 3–6). Furthermore, animals frequently displayed body impacts with the substrate and foot contact errors, including both bumping into the substrate during swing phase and slipping during stance phase (Figs. 3–6). In contrast, the prawns displayed more regular limb movement patterns, though lack of a dorsal camera view precluded full analysis. Since prawn walk with many limbs, the forces and motions can be generated slightly out of phase of each limb, resulting in less variable whole body dynamics and kinematics than if these multiple limbs moved synchronously (as in terrestrial running cockroaches [Full and Tu 1991]). This phenomenon has already been documented in the six walking fin rays of a gurnard by Jamon et al. (2011), in which each fin ray generates a somewhat parabolic reaction force over time, dropping to zero during swing phase. But because the fin rays moved out of phase, the summed force was remarkably consistent (Jamon et al. 2011). We see a similar phenomenon in prawn, with relatively consistent vertical force and velocity over time (Figs. 7 and 9). To attempt to quantify whether prawn were indeed less variable than axolotls, despite the differences in overall length, surface area, and mass, we used coefficient of variation, which expressed the standard deviation within a single trial relative to that trial's mean. These values show that prawn have much less variability than the axolotl in total force magnitude (0.23 vs 0.48, respectively) and direction (0.10 vs 0.34, respectively), and somewhat less variation in total velocity (0.32 vs 0.38, respectively), potentially due to the mitigating effects of a damping environment (Table 1). This more consistent locomotion helps the prawn avoid the seemingly uncontrolled locomotion of the axolotls, characterized by frequent trips, missteps, and body impacts but buffered from consequences by the buoyant support and damping of the surrounding water. However, a high number of legs does not necessarily lead to consistency, as seen in (Martinez et al. 1998), though the consequences of such gaits for variability were not described.

Prior studies of underwater walking have found increased variability in kinematics compared with terrestrial locomotion (Ashley-Ross and Bechtel 2004; Ashley-Ross et al. 2009; Granatosky et al. 2020) however, despite this increased variability, other tetrapods still show a clear underwater gait pattern similar to a running trot (Ashley-Ross and Bechtel 2004, Karakasiliotis et al. 2013). This difference could be due to the neotenic life cycle of axolotls, potentially by removing the constraint of a terrestrial phase of life or by preventing some aspect of maturation. Consistent with the former, lungfish walking underwater show highly variable phase between pelvic fins during underwater walking (King et al. 2011). Additionally, during terrestrial walking, axolotls show very high variability in loading magnitude and timing of footfalls compared with a wide range of other tetrapods (Granatosky et al. 2020). While the need to avoid potentially damaging falls and collisions constrains the gait and mechanics of large, terrestrial animals, buoyancy and drag reduce these consequences, allowing axolotls to use a less constrained gait than terrestrial species of similar sizes and speed (Vogel 1994; Martinez 1996; Vogel 2003). The largest impact force observed for axolotls was 164 mN (Fig. 4, at 1.7–1.8 s), only 14% of the animal's terrestrial body weight. Thus, the fluid environment may insulate underwater walkers from the consequences of locomotor errors in a similar manner to the mechanical feedback seen in small terrestrial insects moving rapidly across rough terrain (Sponberg and Full 2008).

Underwater walking is a remarkably common locomotor behavior, occurring in species with a wide range of body plans, body densities, streamlining, size, life histories, and habitats (Maude and Williams 1983; Denny 1993; Martinez 2001; Vogel 2003; Lim and DeMont 2009; Fletcher et al. 2014). Correspondingly, substrate-based underwater locomotion differs widely in speed, gait, fraction of available limbs used, punting/bounding vs continual substrate contact, and variability of limb and body kinematics (Fish 1987; Ashley-Ross and Bechtel 2004; Ashley-Ross et al. 2009; Lim and DeMont 2009; Macesic and Kajiura 2010; Porter et al. 2022). These differences in morphology and behavior may lead to results which differ from our study taxa. As described above, different taxa can have substantial differences in buoyancy and vertical displacement during underwater locomotion, and consequently can show oscillations of PE more similar in magnitude to KE changes, as seen in our prawns (Zug 1971; Griffin et al. 2004; Peterson and Gomez 2008; Marani et al. 2010; Alam et al. 2015; Withers et al. 2018). Furthermore, substantial interspecific differences in streamlining, such as between slow-moving cryptic taxa (e.g., Antennariid frogfish) (Fish 1987) vs fast-moving taxa (e.g., shore crabs) (Martinez 2001), will result in significant differences in the rate of energy loss to the surrounding water via drag. Differences in overall size will further alter these relationships, due to different scaling relationships between buoyancy, drag, and inertia. Differences in overall body plan (e.g., tetrapods vs decapods) will also affect the above variables (e.g., body density) as well as other features such as the number and type of appendages and the ability to simultaneously use hydrodynamic thrust. Consequently, although all underwater walkers face the same forces, the relative influence of these forces may be very different across taxa, potentially leading to different locomotor strategies.

**Fig. 10:**
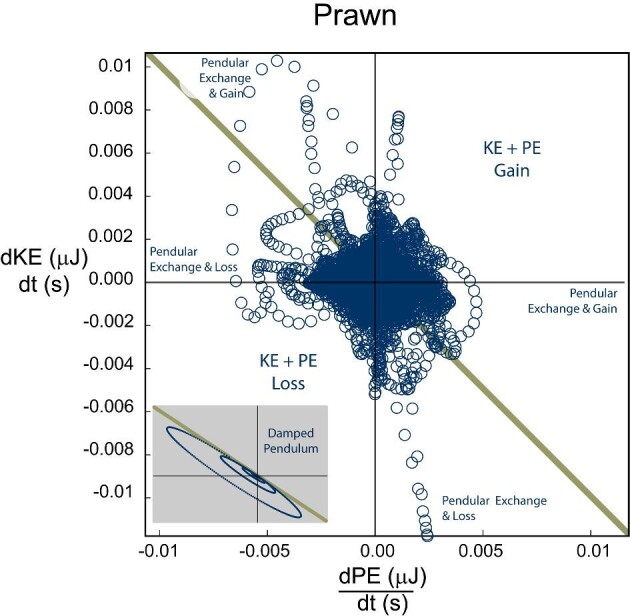
Rates of energy change in Prawn, showing the relationship between the rate of change (power) for potential and KE in all trials of underwater walking in prawns. For a perfect pendular system, the results fall on the dark gold line gold. Despite similar magnitudes of power for both KE and PE, few points fell along the ideal gold line, indicating minimal pendular exchange across all trials. Data points are roughly evenly distributed between, pure loss, pure work, and pendular exchanges with losses or gains. In gray is an example of a dampened pendulum with no energy added to the system.

Thrust generation during aquatic locomotion can come from both imparting momentum to the surrounding fluid, as in swimming, and applying forces to the substrate, as in underwater walking. However, while pelagic swimmers typically lack access to the substrate and thus must rely exclusively on hydrodynamic thrust mechanisms, underwater walkers could hypothetically generate thrust by both mechanisms simultaneously, provided they have suitable anatomy. The prawn used in these experiments have both walking legs and a series of pleopods along the ventral surface of the abdomen, used for pelagic swimming. While the data above is exclusively from trials in which there is no apparent pleopod movement, we do observe trials in which the pleopods are active and generating hydrodynamic forces, with an example of such a trial presented in Fig. 10. During this trial, the prawn shows vertical forces are considerably more variable than typical and with a lower mean vertical force, indicating a vertical component of hydrodynamic thrust, and higher speed despite similar fore-aft forces (Figs. 7 and 10). However, the prawn never fully disengages with the substrate, as evidenced by the continual presence of SRFs, indicating these two methods are indeed applied simultaneously (Figs. 7 and 10). The use of pleopods was also associated by a noticeable snout-down pitch in overall posture, suggesting that the control of two simultaneous sources of thrust poses balance challenges. The use of simultaneous hydrodynamic thrust and propulsive substrate forces opens new possibilities for underwater walking in both animals and robotic systems.

Our results emphasize how differences in the physics of the environment can fundamentally alter the biomechanics of locomotion by removing or mitigating constraints (e.g., minimal consequences for falling due to buoyancy) and imposing new demands and constraints (e.g., the need to overcome drag, loss of effective potential-KE exchange). As a result, behavioral, energetic, and kinetic outcomes are fundamentally different from what is seen in terrestrial environments, which in turn leads to the question of how the dynamics of terrestrial walking evolved from those of underwater walking during the water to land transition. Partial submergence is a literal intermediate between the two environments (water and land), but imposes its own complexities, including reductions in both form and viscous drag and partial buoyant support, as well as additional drag due to surface waves from body and limb motions. Following emergence, belly drag was a likely intermediate behavior (Blob and Biewener 2001; Milàn and Hedegaard 2010; Curth et al. 2014; Farlow et al. 2018; Nyakatura et al. 2019), and allows terrestrial locomotion while potentially displaying broadly similar physics to underwater walking. The support of the ground itself takes the place of buoyancy, precluding effective potential-KE exchange while also eliminating the possibility of falling and impact damage. The resulting frictional forces will oppose motion in a similar manner to drag, albeit without the speed dependence. Consequently, an animal transitioning from underwater walking to belly-dragging animal may be able to achieve effective movement without substantial alterations of their locomotor patterns, thereby easing the evolutionary transition between these tremendously different dynamic environments.

## Supplementary Material

obae013_Supplemental_Files
